# Rapid Transmission of Severe Acute Respiratory Syndrome Coronavirus 2 in Detention Facility, Louisiana, USA, May–June, 2020

**DOI:** 10.3201/eid2702.204158

**Published:** 2021-02

**Authors:** Megan Wallace, Allison E. James, Rachel Silver, Mitsuki Koh, Farrell A. Tobolowsky, Sean Simonson, Jeremy A. W. Gold, Rena Fukunaga, Henry Njuguna, Keith Bordelon, Jonathan Wortham, Melissa Coughlin, Jennifer L. Harcourt, Azaibi Tamin, Brett Whitaker, Natalie J. Thornburg, Ying Tao, Krista Queen, Anna Uehara, Clinton R. Paden, Jing Zhang, Suxiang Tong, Danielle Haydel, Ha Tran, Kaylee Kim, Kiva A. Fisher, Mariel Marlow, Jacqueline E. Tate, Reena H. Doshi, Theresa Sokol, Kathryn G. Curran

**Affiliations:** Centers for Disease Control and Prevention, Atlanta, Georgia, USA (M. Wallace, A.E. James, R. Silver, M. Koh, F.A. Tobolowsky, J.A.W. Gold, R. Fukunaga, H. Njuguna, K. Bordelon, J. Wortham, M. Coughlin, J.L. Harcourt, A. Tamin, B. Whitaker, N.J. Thornburg, Y. Tao, K. Queen, A. Uehara, C.R. Paden, J. Zhang, S. Tong, K. Kim, K.A. Fisher, M. Marlow, J.E. Tate, R.H. Doshi, K.G. Curran);; Louisiana Department of Health, New Orleans, Louisiana, USA (S. Simonson, D. Haydel, H. Tran, T. Sokol)

**Keywords:** severe acute respiratory syndrome coronavirus 2, SARS-CoV-2, coronaviruses, viruses, coronavirus disease, COVID-19, correctional facilities, detention facilities, serial testing, transmission, respiratory infections, zoonoses, Louisiana, United States

## Abstract

To assess transmission of severe acute respiratory syndrome coronavirus 2 (SARS-CoV-2) in a detention facility experiencing a coronavirus disease outbreak and evaluate testing strategies, we conducted a prospective cohort investigation in a facility in Louisiana, USA. We conducted SARS-CoV-2 testing for detained persons in 6 quarantined dormitories at various time points. Of 143 persons, 53 were positive at the initial test, and an additional 58 persons were positive at later time points (cumulative incidence 78%). In 1 dormitory, all 45 detained persons initially were negative; 18 days later, 40 (89%) were positive. Among persons who were SARS-CoV-2 positive, 47% (52/111) were asymptomatic at the time of specimen collection; 14 had replication-competent virus isolated. Serial SARS-CoV-2 testing might help interrupt transmission through medical isolation and quarantine. Testing in correctional and detention facilities will be most effective when initiated early in an outbreak, inclusive of all exposed persons, and paired with infection prevention and control.

Correctional and detention facilities face unique challenges for controlling severe acute respiratory syndrome coronavirus 2 (SARS-CoV-2), the virus that causes coronavirus disease (COVID-19). These challenges include an inability for incarcerated or detained persons to socially distance and an ongoing risk for virus introduction caused by staff movement outside and within the facilities (*1**,**2*). These inherent difficulties underpin increased rates of SARS-CoV-2 infections and deaths among incarcerated and detained persons compared with the general population; 146,472 cases and 1,122 deaths in this population were reported in the United States as of October 20, 2020 (*3**,**4*). The Centers for Disease Control and Prevention (CDC) released interim guidance for management of COVID-19 in correctional and detention facilities; however, some facilities reported limitations to fully implementing the guidance (*5**–**7*). In addition, the potential for asymptomatic and presymptomatic transmission limits the effectiveness of symptom screening to identify cases and halt transmission (*8**–**10*). In other congregate settings, serial testing and physically separating persons based on their SARS-CoV-2 test results have been used to interrupt transmission (*11**,**12*).

We investigated a COVID-19 outbreak in a detention center in Louisiana, USA (facility X) and used a serial testing strategy to identify infections and interrupt transmission in affected dormitories. All residents of affected dormitories underwent SARS-CoV-2 testing to assess the extent of transmission within the dormitory, to cohort detained persons based on their test result to prevent transmission, and to evaluate the utility of serial testing in this setting. We report the findings of this investigation; initial results were previously reported (*13*).

By March 17, 2020, in response to emergence of COVID-19 in Louisiana, facility X ceased travel of detained persons outside the facility, halted visitors and transfers between facilities, and prohibited movement of detained persons within the facility. On March 29, a staff member showed symptoms consistent with COVID-19; this staff member later tested positive for SARS-CoV-2. On April 7, facility X medical staff identified the first COVID-19 case in a detained person residing in dormitory A. After this diagnosis, staff began active daily monitoring for fever (temperature >100.4°F) and blood oxygen saturation levels (pulse oximeter reading <90%) to detect suspected cases among persons in affected dormitories. On April 9, additional cases were identified in dormitories B and C; the first cases were identified in dormitory D on April 17 and in dormitory E on April 23.

The Louisiana Department of Health requested CDC assistance; a team arrived and began an investigation on May 7. By that date, 3 staff members and 35 detained persons showed development of symptoms and later tested positive for SARS-CoV-2; 5 of 18 dormitories were affected.

## Methods

### Population

Facility X is a medium-security local jail that houses up to 800 detained persons. Before the COVID-19 pandemic, the facility operated at nearly 100% capacity. On May 7, the facility was at ≈85% capacity because of a reduction in occupancy in response to COVID-19. Detained persons from 6 dormitories (A–F) were enrolled in this prospective cohort investigation. Five dormitories (A–E) had detained persons with laboratory-confirmed COVID-19 cases; dormitory F, which housed a detained person with COVID-19 symptoms and negative SARS-CoV-2 test results, was enrolled because of proximity to dormitories A, B, and D. All detained persons with suspected and confirmed COVID-19 were moved to medical isolation, and persons within the dormitories were quarantined as a cohort.

### Testing Strategy and Cohorting by Test Result

**Figure 1:**
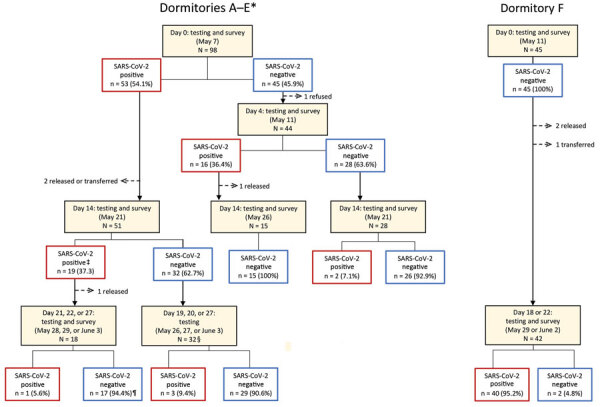
Rapid transmission of SARS-CoV-2 in detention facility, Louisiana, USA, May–June 2020. Enrollment and follow-up at each timepoint for detained persons (n = 143) in dormitories A–E and F. The sequence of testing for all enrolled dormitories is shown, along with the number of persons who were positive or negative for SARS-CoV-2 by real-time reverse transcription PCR and percentage of total at each timepoint. Red boxes indicate SARS-CoV-2 positive, and blue boxes indicate SARS-CoV-2 negative. *The first positive test result for SARS-CoV-2 among persons detained occurred on the following dates in each dormitory: April 7 in A, April 9 in B and C, April 17 in D, and April 23 in E. Introduction in dormitory F likely occurred between May 11 and May 29. †One inconclusive result was considered negative; ‡One inconclusive result was considered positive. §16 persons were tested on May 26 only, 14 on May 27 only, and 2 on May 26 and June 3. ¶10 persons were tested on May 28 only, 1 on May 29 only, 1 on June 3 only, and 6 on May 28 and June 3. SARS-CoV-2, severe acute respiratory syndrome coronavirus 2.

Nasopharyngeal swab specimens were collected for initial SARS-CoV-2 testing on day 0 for all consenting persons residing in dormitories A–F (Figure 1). Persons who had positive results by real-time reverse transcription PCR (rRT-PCR) were moved to the designated SARS-CoV-2–positive dormitories upon facility receipt of results (<24 hours after specimen collection). Serial testing was offered on day 4 to detained persons who tested negative for SARS-CoV-2 on day 0, and again on day 14 for persons who tested negative on day 4. To assess persistence of viral shedding, detained persons testing positive on day 0 or day 4 were offered testing 14–15 days and 19–27 days after their first positive test result.

In dormitory F, where all detained persons tested negative for SARS-CoV-2 on day 0, a serial testing strategy was not used. Rather, a second survey and repeat test was conducted on day 18.

### Dormitory Survey and Symptoms, Concurrent Conditions, and Behavioral Risk Assessment

The investigation team administered a structured dormitory survey among facility staff to assess physical layout, capacity, activities, and practices. During day 0 testing, detained persons completed a self-administered, paper-based questionnaire of demographics, symptoms in the preceding 2 months and 2 weeks, facility exposures, and preventive measures. On the day of each subsequent test, detained persons received an abbreviated self-administered, paper-based questionnaire of symptoms experienced since the last testing day. The team verbally verified responses with detained persons and assisted as necessary. Medical history data were abstracted from facility medical records. Data were deidentified and entered into a secure database (Research Electronic Data Capture, version 8.8.0; Vanderbilt University, https://redcap.vanderbilt.edu).

### Laboratory Testing

Nasopharyngeal swab specimens collected for the investigation during May 7–June 3 were immediately placed on dry ice and sent by courier to the Louisiana Office of Public Health Laboratory for SARS-CoV-2 testing by using the CDC 2019-Novel Coronavirus (2019-nCoV) Real-Time rRT-PCR Diagnostic Panel. Cycle threshold (C_t_) values for 2 viral nucleocapsid protein genes (N1 and N2) were obtained for each specimen; C_t_ values <40 cycles for both N1 and N2 were considered positive for SARS-CoV-2 (*14*). All samples that were positive at the Louisiana Office of Public Health Laboratory were refrozen and shipped to CDC for viral culture by using Vero-CCL-81 cells (*15*). Positive viral culture for SARS-CoV-2 replication-competent virus was confirmed in cells that showed a cytopathic effect by using rRT-PCR.

Nucleic acid was extracted from 41 rRT-PCR–positive specimens or isolates and subjected to Oxford Nanopore MinION Sequencing (https://nanoporetech.com) according to published protocols (*16*); consensus sequences were generated by using Minimap version 2.17 (https://github.com/lh3/minimap2) and Samtools version 1.9 (http://www.htslib.org). Representative full-genome sequences were downloaded on August 28, 2020, from GISAID (https://www.gisaid.org), and phylogenetic relations were inferred by using maximum-likelihood analyses implemented in TreeTime (https://evol.bio.lmu.de/_statgen/software/treetime) and the Nextstrain pipeline (*17*). Sequences were submitted to GenBank and GISAID.

### Analyses

We performed descriptive analyses for the population demographics (age, sex, race/ethnicity), underlying medical conditions (respiratory disease, diabetes, hypertension, other cardiovascular disease, other condition), obesity (body mass index >30 kg/m^2^), tobacco use, and dormitory characteristics (capacity at start of the investigation, toilets/sinks, showers per person). Overall cumulative incidence and dormitory cumulative incidence for each test day were calculated.

We calculated descriptive statistics for C_t_ values and culture results, stratified by symptom status. The rRT-PCR analyses used the C_t_ value reported for the N1 genetic target because N1 and N2 approximate each another (*18*). Persons were categorized as presymptomatic, symptomatic, postsymptomatic, or asymptomatic on the basis of symptoms at sample collection. Any CDC-listed coronavirus symptom with a reported onset date on or after March 29, 2020, the illness onset date of the first reported COVID-19 case in the facility, was included in analyses (*19*). Persons were classified as symptomatic if they reported >1 present or ongoing symptom. If 2 courses of illness were distinguishable from the symptom data, in which multiple symptoms were reported to occur with symptom onsets >14 days apart and the first course of illness (earlier dated symptoms) was reported to have resolved, only the symptoms reported closer to the date of testing were used for classification. Postsymptomatic persons were those who reported symptoms that had resolved before the first positive test result or before the start of the investigation (day 0) for those who were tested and remained negative during the investigation. Persons reporting symptoms whose surveys were missing current symptom status were considered symptomatic if the onset date was <10 days the start of the investigation. Presymptomatic persons reported >1 symptom with onset after their first positive test result and had no previously reported symptoms. Asymptomatic persons reported no symptoms throughout the investigation. Persons were classified as having an unknown symptom status if any symptom data were missing and no symptoms were reported. C_t_ value and culture results were graphed by days from symptom onset and original dormitory.

To compare individual symptoms, facility exposures (bunk sleeping location, travel out of dormitory, exposure to someone visibly ill), and preventive measures (handwashing, mask use) by SARS-CoV-2 test result, we performed bivariate analyses by using Fisher exact tests for proportions. Analyses were completed by using R statistical software version 4.0.0 (The R Foundation, https://www.r-project.org) and SAS 9.4 software version 6.2.92 (SAS Institute Inc., https://www.sas.com). 

### Ethics

This activity was determined to meet the requirements of public health surveillance as defined in 45 CFR 46.102(l) (*2*). All persons provided voluntary oral consent for testing and to complete questionnaires.

## Results

### Dormitory and Detained Persons Characteristics

All 143 detained persons from 6 dormitories were invited for testing, and 143 (100%) participated in the day 0 testing and survey (Figure 1). Median age was 33 (interquartile range 28–42) years, and most (136, 95%) were male (Table 1). Most (102, 71%) were Black non-Hispanic persons, and 36 (25%) were White non-Hispanic persons. One third (49, 34%) of the 143 detained persons had an underlying medical condition. Dormitory E was the only female dormitory. Dormitory C had the highest median age (45 years; interquartile range 35–52 years) and the highest proportion (7/11; 64%) of persons with underlying medical conditions. Dormitory E had the lowest percent occupancy (7/22; 32%), whereas dormitory F was near full capacity (45/50; 90%). All dormitories had 3–4 shared toilets and sinks and 2–3 shared showers.

**Table 1:** Characteristics of detained persons tested for SARS-CoV-2 in a correctional facility, Louisiana, USA, by dormitory, May–June 2020*

Characteristic	Dormitory A, n = 20	Dormitory B, n = 23	Dormitory C, n = 11	Dormitory D, n = 37	Dormitory E, n = 7	Dormitory F, n = 45	Total, N = 143
Median age, y (IQR)	37 (29–47)	31 (29–36)	45 (35–52)	31 (29–39)	37 (29–47)	32 (24–41)	33 (28–42)
Sex							
M	20 (100)	23 (100)	11 (100)	37 (100)	0	45 (100)	136 (95)
F	0	0	0	0	7 (100)	0	7 (5)
Race/ethnicity							
White non-Hispanic	10 (50)	6 (26)	7 (64)	5 (14)	2 (29)	5 (11)	36 (25)
Black non-Hispanic	10 (50)	16 (70)	4 (36)	30 (81)	5 (71)	37 (82)	102 (71)
Asian non-Hispanic	0	0	0	1 (3)	0	0	1 (1)
Hispanic/Latino	0	0	0	1 (3)	0	3 (8)	4 (3)
Underlying health condition							
Any	8 (40)	7 (30)	7 (64)	14 (38)	3 (43)	10 (22)	49 (34)
Respiratory disease	3 (15)	3 (13)	3 (27)	5 (14)	1 (14)	3 (7)	18 (13)
Asthma	1 (5)	1 (4)	3 (27)	4 (11)	0	3 (7)	12 (8)
Diabetes	1 (5)	0	3 (27)	0	2 (29)	1 (2)	7 (5)
Hypertension	3 (15)	3 (13)	5 (45)	7 (19)	2 (29)	7 (15)	27 (19)
Other CVD	0	1 (4)	0	2 (5)	0	1 (2)	4 (3)
Other†	4 (15)	2 (8)	1 (9)	2 (5)	0	1 (2)	10 (7)
Obesity, BMI >30 kg/m^2^	6 (30)	7 (30)	1 (9)	7 (19)	2 (29)	6 (13)	29 (20)
Any past tobacco use	12 (60)	5 (22)	8 (73)	14 (38)	4 (57)	17 (38)	60 (42)
Dormitory							
Capacity at start of study	20/30 (67)	23/30 (77)	11/22 (50)	37/50 (74)	7/22 (32)	45/50 (90)	NA
Toilets/sinks	3	3	4	3	4	3	NA
Showers/person	3	3	2	3	2	2	NA

*Values are no. (%) or no. unless indicated otherwise. BMI, body mass index; CVD, cardiovascular disease; IQR, interquartile range; NA, not applicable; SARS-CoV-2, severe acute respiratory syndrome coronavirus 2. †Includes liver disease, immunosuppressive disorder, and neurologic disease.

### Serial Testing

In dormitories A–E, 53 (54%) persons tested positive on day 0 (Table 2). Among persons with negative test results from day 0 testing in dormitories A–E (n = 45), 16 (36%) had SARS-CoV-2 detected on day 4 testing. Two additional persons tested positive for SARS-CoV-2 on day 14, both of whom originally resided in dormitory B. No SARS-CoV-2 infections (0/45) were detected during the day 0 testing in dormitory F. However 40 (89%) of 45 persons tested positive for SARS-CoV-2 on day 18. No detained persons testing positive for SARS-CoV-2 from any dormitory required hospitalization during their illness.

**Table 2:** Cumulative incidence of SARS-CoV-2 infection in 143 detained persons by time point and original dormitory in a correctional facility, Louisiana, USA, May–June, 2020*

Dormitory	Days since first positive test result for SARS-CoV-2					Cumulative incidence by dormitory and overall, no. positive/no. tested (%)
		SARS-CoV-2 positive, no. (%)				
		Day 0	Day 4	Day 14	Day 18	
A, n = 20	30	13/20 (65)	2/7 (29)	0/5 (0)	NA	15/20 (75)
B, n = 23	28	10/23 (43)	4/13 (31)	2/9 (22)	NA	16/23 (70)
C, n = 11	28	6/11 (55)	3/5 (60)	0 /2 (0)	NA	9/11 (82)
D, n = 37	20	20/37 (54)	7/16 (44)	0/10 (0)	NA	27/37 (73)
E, n = 7	14	4/7 (57)	0/3 (0)	0/3 (0)	NA	4/7 (57)
F, n = 45	Unknown†	0/45 (0)	NA	NA	40/45 (89)	40/45 (89)
Cumulative incidence by day		53/143 (37)	16/44 (36)	2/29 (7)	40/45 (89)	111/143 (78)

*NA, not applicable; SARS-CoV-2, severe acute respiratory syndrome coronavirus 2. †Introduction in dormitory F occurred at some point between day 0 and day 18.

The overall cumulative incidence during May 7–June 3 of SARS-CoV-2 infection for all dormitories was 78% (111/143). Dormitory E had the lowest cumulative incidence (57%; 4/7), and dormitory F had the highest cumulative incidence (89%; 40/45). Day 0 testing in dormitory E was initiated 14 days after the diagnosis of the first known COVID-19 case in the dormitory, and dormitories A–D had reported cases 20–30 days before the investigation.

Of 111 detained persons with SARS-CoV-2-positive test results, 66 persons received a second test (day 14) and 50 people received a third test (during days 19–27) during the investigation (Figure 1). Nineteen (29%) of 66 persons had positive test results 14 days after the first positive test result, and 4 (8%) of 50 persons had positive test results ≈3 weeks after first testing positive, 3 of whom had negative results on day 14.

### Symptom and Behavioral Risk Assessment

Among 111 detained persons who tested positive for SARS-CoV-2, 21 (19%) were symptomatic at the time of their first positive test result, and 27 (24%) reported symptoms that had resolved before their first positive test result (Table 3). The most commonly reported symptoms among persons with SARS-CoV-2 infection were headache (32%), loss of taste or smell (31%), and nasal congestion (26%); measured fever (5%) and dyspnea (8%) were less commonly reported (Appendix Table 1). Forty-nine (44%) detained persons who tested positive for SARS-CoV-2 were asymptomatic and 3 (3%) were presymptomatic. Symptom onset among presymptomatic persons was 0–7 days from the day of first positive specimen collection. Among 32 detained persons with negative test results, 8 (25%) were symptomatic and 9 (28%) were postsymptomatic. No enrolled detained persons were hospitalized or died. No major differences in handwashing practices, mask use, and movement within the facility were reported by those who tested positive compared with those who tested negative (Appendix Table 2).

**Table 3:** Symptom status of 143 detained persons at time of testing for SARS-CoV-2 and throughout course of investigation in a correctional facility, Louisiana, USA, May–June 2020*

Symptom status†	SARS-CoV-2 testing results from first positive test result			SARS-CoV-2 negative, no. (%)
	SARS-CoV-2 positive, no. (%)	Median C_t_ values (range)‡	Culture positive, no. (%)§	
Presymptomatic	3 (3)	30.6 (20.0–31.1)	2 (8)	NA
Symptomatic	21 (19)	32.7 (19.7–36.3)	6 (24)	8 (25)
Postsymptomatic	27 (24)	33.2 (25.2–37.5)	3 (12)	9 (28)
Asymptomatic	49 (44)	32.9 (19.8–36.9)¶	12 (48)#	12 (34)
Unknown	11 (10)	33.1 (25.1–35.7)	2 (8)	3 (9)
Overall	111 (78)	33 (19.7–37.5)	25 (23)	32 (22)

*SARS-CoV-2 testing was conducted by using the Centers for Disease Control and Prevention 2019-Novel Coronavirus (2019-nCoV) Real-Time RT-PCR Diagnostic Panel. The C_t_ values reported for nucleocapsid protein gene 1 target are shown. C_t_, cycle threshold; NA, not applicable; SARS-CoV-2, severe acute respiratory syndrome coronavirus 2. †Symptom status at time of first positive test result or throughout the investigation for persons remaining SARS-CoV-2 negative. Presympomatic: at least 1 symptom started after positive test result and no symptoms before positive test result; symptomatic: at least 1 symptom ongoing at time of test result (first positive, or any negative test result); postsymptomatic: at least 1 symptom started before test result (first positive result) or before investigation start date (continuous negative results); asymptomatic: no symptoms before test result (first positive result or before each negative test result); unknown: at least 1 symptom is unknown during at least 1 interview. Symptoms assessed: fever, subjective fever, cough, shortness of breath, chills, myalgia, sore throat, loss of taste or smell, or diarrhea ‡Tukey’s test for significance, p = 0.03. §Viral culture positive for replication-competent virus. ¶One person missing a C_t_ value on the initial day this person tested positive. #One specimen from an asymptomatic person who was positive by real-time reverse transcription PCR was not submitted for culture.

### C_t_ Values and Viral Culture

**Figure 2:**
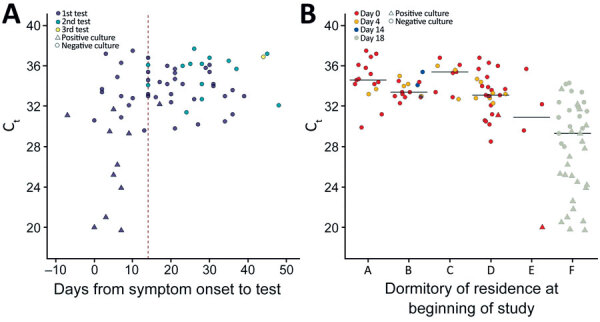
Rapid transmission of severe acute respiratory syndrome coronavirus 2 (SARS-CoV-2) in detention facility, Louisiana, USA, May–June 2020. A) C_t_ values and viral culture results by days from symptom onset of any symptom in SARS-CoV-2–positive detained persons. Nucleocapsid protein 1 target C_t_ values and viral culture results of 66 specimens from 51 persons who were positive for SARS-CoV-2 by days from reported symptom onset. C_t_ values and viral culture results are also shown for 14 of the 51 specimens from persons who were positive a second time, and for 1 specimen that remained positive for a third test. Vertical dashed line indicates day 14 to depict the recommended medical isolation timeframe from symptom onset for persons in congregate settings. Shapes indicate culture results, and colors indicate day of positive test result. One positive test result is not included because C_t_ values were not reported. B) C_t_ values and viral culture results for SARS-CoV-2–positive detained persons at the time of first sample collection according to dormitory residence and day of first positive result. Nucleocapsid protein 1 target C_t_ values and viral culture results of the first SARS-CoV-2–positive test result for 110 detained persons is shown by dormitory of residence at the time of first sample collection. Horizontal lines indicate median C_t_ values for first positive samples from residents in each dormitory. One positive test result from a dormitory F resident is not included because C_t_ value was not reported. C_t_ , cycle threshold.

Median C_t_ values were lowest among presymptomatic persons (30.6, range 20.0–31.1) and highest among postsymptomatic persons (33.2, range 25.2–37.5) (p = 0.03). The overall ranges for C_t_ values were similar for symptomatic (19.7–36.3) and asymptomatic persons (19.8–36.9). Among the 51 symptomatic SARS-CoV-2–positive persons, positive rRT-PCR results occurred 7 days before symptom onset to 48 days after symptom onset (Figure 2, panel A).

Among 111 specimens that resulted in the first positive results for detained persons, 110 were submitted for viral culture and 25 (23%) had replication-competent virus isolated (Table 3). Replication-competent virus isolates were obtained from 25% (12/48) of nasopharyngeal swab specimens from asymptomatic persons, 67% (2/3) from presymptomatic persons, 29% (6/21) from symptomatic persons, and 11% (3/27) from postsymptomatic persons. Among persons reporting symptoms, specimens with replication-competent virus were collected during 6 days before to 4 days after symptom onset. Two postsymptomatic persons reported symptom resolution the day of testing; for the third person, date of symptom resolution was unknown.

The C_t_ values at the first positive test result and the proportion of specimens with positive viral culture for SARS-CoV-2 varied by dormitory (Figure 2, panel B). The median C_t_ value for 53 specimens collected from detained persons in dormitories A–E was 33.6 (range 20.0–37.5); 2 (4%) samples from persons in dormitories D and E were replication competent. The median C_t_ value for 39 samples from detained persons in dormitory F was 29.3 (range 19.7–34.3). Of these samples, 23 (59%) were replication competent.

Of 22 persons that had positive test results >14 days after the first positive test, 4 remained rRT-PCR positive for SARS-CoV-2 ≈3 weeks after first testing positive. Virus isolation was attempted but was not successful for any of the specimens from repeat-positive persons.

### Phylogenetic Analysis

We compared sequencing results for 41 specimens collected from persons in dormitories A (n = 2), D (n = 5), E (n = 2), and F (n = 32) at facility X during May 7–29 with each other and representative sequences from GISAID. All sequences clustered together within clade 20C and among other sequences reported from Louisiana (Appendix Figure). A phylogenetic tree illustrated 3 groups: 1 with sequences from persons in dormitories D and E, a second with sequences from persons in dormitories A and D, and a third with sequences from persons in dormitory F. Two identical SARS-CoV-2 sequences were identified from a person in dormitory D and a person from dormitory E. The third group differed from the first cluster by >6 nt and from the second cluster by 2 nt mutations.

## Discussion

Through serial testing of detained persons from quarantined dormitories at a Louisiana detention facility, we identified rapid and widespread SARS-CoV-2 transmission, a large number of asymptomatic infections, and shedding of replication-competent virus in persons with asymptomatic and presymptomatic infections. Despite early adoption of certain prevention and mitigation measures, the cumulative incidence among affected dormitories in facility X was 78%. Of persons who tested positive for SARS-CoV-2, 47% (52/111) were asymptomatic, of which 12 had positive viral culture results with replication-competent virus, indicating infectiousness. In this relatively young population, C_t_ values were similar regardless of symptom status; the lowest C_t_ values were among persons with presymptomatic infection, indicating high viral load (*20*). These findings add to the evidence that presymptomatic and asymptomatic persons can transmit SARS-CoV-2 (*8*).

This investigation demonstrated the usefulness of testing shortly after SARS-CoV-2 introduction and at multiple time points to comprehensively identify infections and mitigate transmission. Serial testing identified 52% (58/111) of the COVID-19 cases identified during the investigation. In dormitories A–E, 2 of 53 positive samples from day 0 testing had replication-competent virus, suggesting many persons in these dormitories were convalescent. In dormitory F, 89% (40/45) of residents tested positive for SARS-CoV-2 18 days after all testing negative on day 0; 59% had replication-competent virus. The timing of initial testing in dormitories A–E (2–4 weeks after the first case) and the long testing interval (18 days) in dormitory F limited the usefulness of serial testing to provide data needed to mitigate transmission. Once SARS-CoV-2 introduction into a correctional or detention facility is suspected or confirmed, widespread testing of detained persons and staff at short intervals could quickly identify infections and inform cohorting by infection status to prevent further transmission. In nursing homes, facilitywide testing closer in time to the identification of a COVID-19 case was associated with fewer cases within the facility (*21*). Facilities with resource constraints for which widespread testing is not feasible should work with the local health department to determine the most effective testing strategy for their facility.

To complement symptom screening and address the challenges of early detection of SARS-CoV-2, correctional and detention facilities might consider both periodic testing at regular intervals (e.g., 7–14 days) and serial testing of close contacts at short intervals (e.g., 3–4 days) to identify newly acquired infections, infections missed in previous rounds of testing, and new introductions (*8**,**12**,**20*). Increased dormitory density might also be a risk factor for viral transmission; the lowest cumulative incidence occurred in dormitory E, which had lowest occupancy. Some facilities have reduced occupancy as a mitigation strategy (*6*). Novel testing approaches (e.g., pooled testing), point-of-care rapid antigen assays, and less intrusive specimen collection methods are urgently needed to enable efficient SARS-CoV-2 testing. This investigation found no differences in handwashing and mask use between persons who tested positive or negative for SARS-CoV-2. A small proportion overall (13%) reported always using a mask which, along with close living quarters, might have limited the effectiveness of these personal mitigation measures.

During follow-up, 22 persons tested positive ≥14 days after their first positive result and 1 person tested positive 48 days after symptom onset. Four persons had positive rRT-PCR results ≈3 weeks after the first positive result, which was longer than that seen in previous investigations of patients with mild illness (*22**,**23*). However, replication-competent virus was not isolated from these specimens or any specimens collected >9 days after symptom onset. This finding lends support to facilities using symptom-based criteria for release after 10 days of isolation, with resolution of fever and improvement of other symptoms, instead of test-based criteria (*24*).

Phylogenetic analysis identified 3 distinct clusters of SARS-CoV-2 infection from 41 specimens collected within the same month from detained persons in dormitories A, D, E, and F. Given the genetic distance between the groups within a short time period and the overall diversity of sequences from the COVID-19 outbreak, there was likely >1 introduction of SARS-CoV-2 into the facility before May 29. In addition to mitigation measures to prevent SARS-CoV-2 spread within a facility, measures should be taken to limit introductions into the facility, including routine symptom screening and testing at entry, use of face masks, and systematic assignment of staff to specific dormitories.

We identified 4 primary limitations to this investigation. First, serial testing was initiated 2–4 weeks after the first case was identified in dormitories A–E, which limited our ability to assess the impact of testing and cohorting on preventing transmission if most detained persons had been infected before the investigation. In addition, persons who tested negative for SARS-CoV-2, including 53% who reported COVID-19 symptoms, might have had COVID-19 and cleared their infections by the time of testing, leading to an underestimation of the prevalence of SARS-CoV-2 infection. No antibody testing was performed; thus, the extent of prior infection cannot be estimated. Second, detained persons might have limited recall of mild symptoms and symptom timing, particularly symptoms occurring >2 weeks before testing, potentially resulting in an overestimation of the prevalence of asymptomatic infection. Also, follow-up symptom assessments were not conducted among persons with positive test results from dormitory F, thus potential presymptomatic detained persons remained classified as asymptomatic. Third, given our inclusion of symptoms reported up to 6 weeks before testing, misclassification of symptoms caused by other pathogens or allergies could have occurred. Finally, no systematic testing of facility staff or detained persons in other dormitories was part of this investigation.

In correctional and detention facilities, prevention and mitigation of SARS-CoV-2 transmission requires a combination of measures (*5*). Testing is necessary to identify asymptomatic and presymptomatic persons who can silently transmit the infection. Although symptom screening alone was not sufficient to identify SARS-CoV-2 infections, it could serve as a signal for SARS-CoV-2 introduction and initiation of widespread testing. To increase sensitivity of symptom screening, screenings should use an expanded COVID-19 symptom list based on the latest evidence and guidance, and barriers to symptom reporting, such as medical care costs or concerns over medical isolation, should be minimized (*18**,**25**,**26*). Multiple rounds of widespread testing for detained persons and staff might be necessary for early detection of virus introduction, particularly when there are high rates of transmission in the surrounding community and ongoing risk for reintroduction. When initiated early in an outbreak, results from serial testing 3–4 days after an exposed person first tests negative for SARS-CoV-2, paired with mitigation strategies, might help limit transmission among detained persons. SARS-CoV-2 testing in these congregate settings will likely be most effective when timed soon after viral introduction, inclusive of all potentially exposed staff and detained persons, and combined with infection control mitigation strategies such as medical isolation and quarantine.

Appendix Additional information on rapid transmission of severe acute respiratory syndrome coronavirus 2 in detention facility, Louisiana, USA, May–June, 2020.
