# Supplementary material for: Rapid Transmission of Severe Acute Respiratory Syndrome Coronavirus 2 in Detention Facility, Louisiana, USA, May–June, 2020
Source: Emerg Infect Dis. 2021 Feb;27(2):421–9. doi: 10.3201/eid2702.204158 (PMC7853536; doi:10.3201/eid2702.204158)
Supplement: Appendix — Additional information on rapid transmission of severe acute respiratory syndrome coronavirus 2 in detention facility, Louisiana, USA, May–June, 2020. [file 20-4158-Techapp-s1.pdf]

Article DOI: https://doi.org/10.3201/eid2702.204158

Rapid Transmission of Severe Acute Respiratory Syndrome Coronavirus 2 in Detention Facility, Louisiana, USA, May–June, 2020

Appendix

Appendix Table 1. Symptoms reported by 143 detained persons throughout course of investigation by SARS-CoV-2 test status, Louisiana, USA, May–June, 2020*

Symptom	Overall, n = 143	SARS-CoV-2 positive, n = 111†	SARS-CoV-2 negative, n = 32	p value‡
Symptomatic (at any point)§	68 (48)	51 (46)	17 (53)	0.87
Headache	46 (32)	35 (32)	11 (34)	0.83
Loss of taste or smell	43 (30)	34 (31)	9 (28)	0.83
Nasal congestion	37 (26)	29 (26)	8 (25)	1.00
Rhinorrhea	28 (20)	21 (19)	7 (22)	0.80
Subjective fever	24 (17)	5 (16)	19 (17)	1.00
Chills	24 (17)	18 (16)	6 (19)	0.79
Myalgia	24 (17)	19 (17)	5 (16)	1.00
Cough	20 (14)	15 (14)	5 (16)	0.78
Abdominal pain	19 (13)	15 (14)	4 (13)	1.00
Diarrhea	17 (12)	14 (13)	3 (9)	0.76
Dyspnea	15 (11)	9 (8)	6 (19)	0.10
Sore throat	14 (10)	11 (10)	3 (9)	1.00
Nausea	13 (9)	8 (7)	5 (16)	0.17
Vomiting	6 (4)	6 (5)	0 (0)	0.21
Measured fever	5 (4)	5 (5)	0 (0)	0.59

*Values are no. (%). SARS-CoV2, severe acute respiratory syndrome coronavirus 2.
†SARS-CoV-2 testing was conducted by using the Centers for Disease Control and Prevention 2019-Novel Coronavirus (2019-nCoV) Real-Time Reverse Transcription PCR Diagnostic Panel.
‡By Fisher exact test.
§Inclusive of presymptomatic, symptomatic, and postsymptomatic persons who reported symptoms at any time during the investigation.

Appendix Table 2. Facility exposures reported by 143 detained persons on day 0 of investigation by SARS-CoV-2 test results, Louisiana, USA, May–June, 2020*

Reported facility exposures at 6 dormitories	Total, N = 143	SARS-CoV2 positive, n = 111 (78%)	SARS-CoV2 negative, n = 32 (78%)	p value†
Risk factor				
Sleeping location bottom bunk	90 (63)	71 (64)	19 (59)	0.68
Wash hands				
Before eating	100 (70)	78 (70)	22 (69)	1.00
After touching a shared phone	107 (75)	85 (77)	22 (69)	0.37
After coughing or sneezing	98 (69)	76 (68)	22 (69)	1.00
After touching another person	77 (54)	58 (52)	19 (59)	0.55
After using the bathroom	131 (92)	100 (90)	31 (97)	0.30
After touching dirty laundry	87 (61)	68 (62)	19 (59)	0.84
After working	45 (31)	34 (31)	11 (34)	0.67
Never	1 (1)	1 (1)	0 (0)	1.00
Worn mask in past 2 weeks	111 (78)	85 (77)	26 (81)	0.20
Wear mask when around others				0.76
Always	18 (13)	12 (11)	6 (19)	NA
Usually	20 (14)	16 (14)	4 (13)	NA
Sometimes	62 (43)	48 (43)	14 (44)	NA
Never	11 (8)	9 (8)	2 (6)	NA
Mask type				
Surgical	35 (24)	267(24)	(25)	1.00
Cloth	48 (34)	41 (37)	7 (22)	0.13
Travel out of dormitory	109 (76)	82 (74)	27 (84)	0.34
Around anyone visibly ill	61 (43)	44 (40)	17 (53)	0.42

*Values are no. (%). NA, not applicable; SARS-CoV2, severe acute respiratory syndrome coronavirus 2.
†By Fisher exact test.

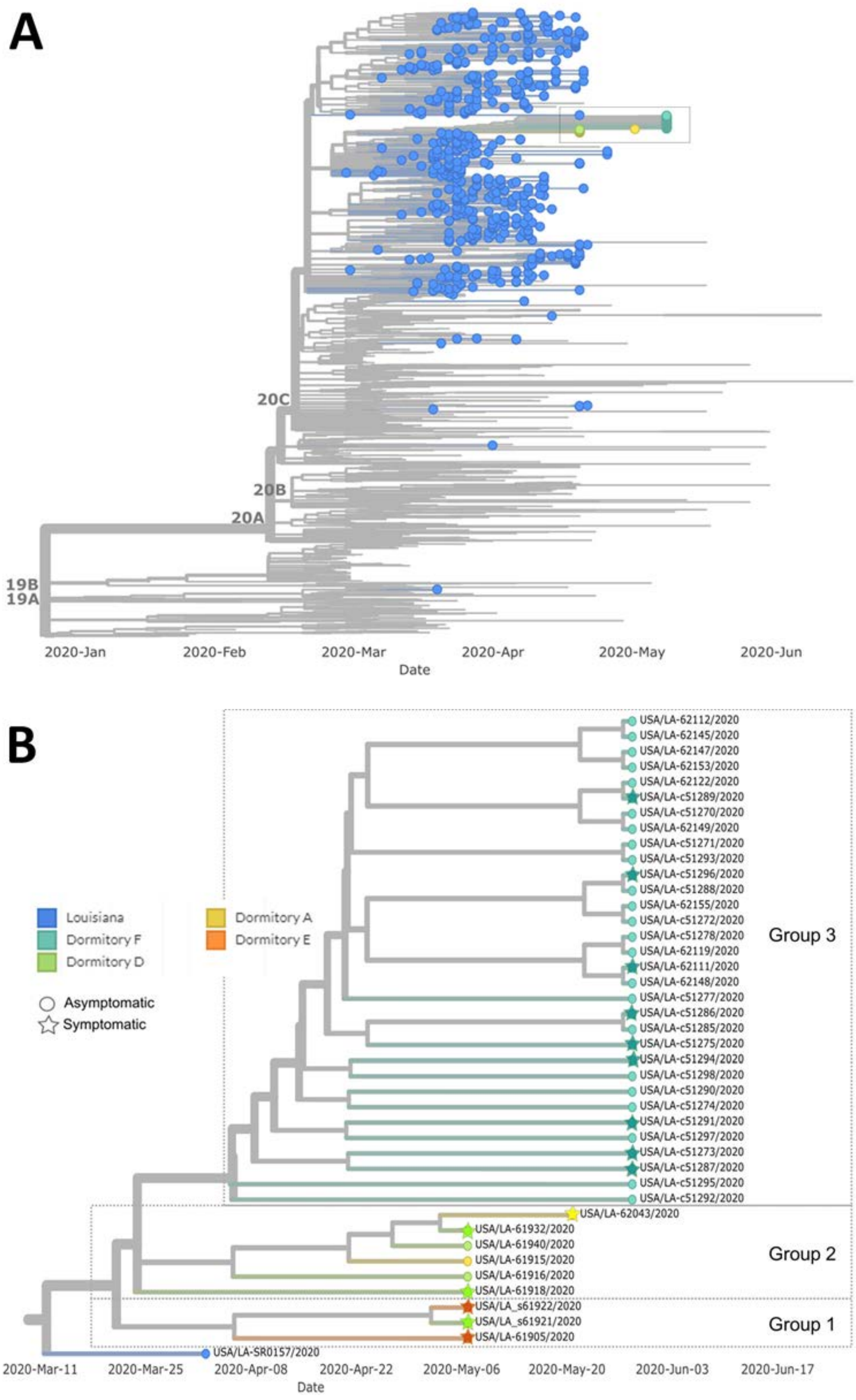
A
20C
20B
20A
19B
19A
2020-Jan
2020-Feb
2020-Mar
2020-Apr
2020-May
2020-Jun
Date
B
USA/LA-62112/2020
USA/LA-62145/2020
USA/LA-62147/2020
USA/LA-62153/2020
USA/LA-62122/2020
USA/LA-c51289/2020
USA/LA-c51270/2020
USA/LA-62149/2020
USA/LA-c51271/2020
USA/LA-c51293/2020
USA/LA-c51296/2020
USA/LA-c51288/2020
USA/LA-62155/2020
USA/LA-c51272/2020
USA/LA-c51278/2020
USA/LA-62119/2020
USA/LA-62111/2020
USA/LA-62148/2020
USA/LA-c51277/2020
USA/LA-c51286/2020
USA/LA-c51285/2020
USA/LA-c51275/2020
USA/LA-c51294/2020
USA/LA-c51298/2020
USA/LA-c51290/2020
USA/LA-c51274/2020
USA/LA-c51291/2020
USA/LA-c51297/2020
USA/LA-c51273/2020
USA/LA-c51287/2020
USA/LA-c51295/2020
USA/LA-c51292/2020
Group 3
Louisiana
Dormitory F
Dormitory D
Dormitory A
Dormitory E
Asymptomatic
Symptomatic
USA/LA-62043/2020
USA/LA-61932/2020
USA/LA-61940/2020
USA/LA-61915/2020
USA/LA-61916/2020
USA/LA-61918/2020
Group 2
USA/LA_s61922/2020
USA/LA_s61921/2020
USA/LA-61905/2020
Group 1
USA/LA-SR0157/2020
2020-Mar-11
2020-Mar-25
2020-Apr-08
2020-Apr-22
2020-May-06
2020-May-20
2020-Jun-03
2020-Jun-17
Date

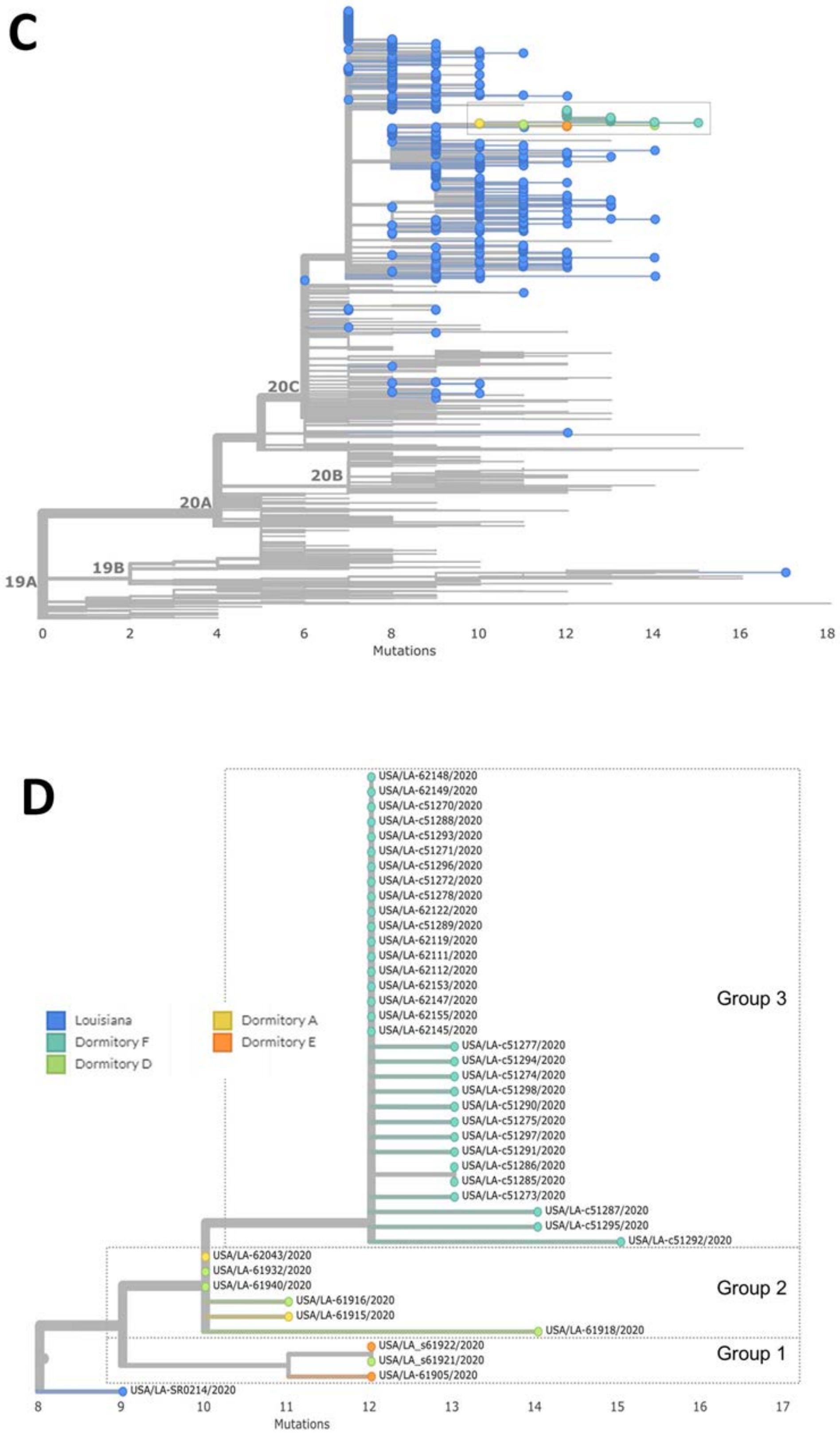

Appendix Figure. Phylogenetic trees with branch views depicting divergence over time (A and B) and by single-nucleotide mutations (C and D) of 41 severe acute respiratory syndrome coronavirus 2 genomes obtained from detainees in Facility X, Louisiana, USA, May–June, 2020. A and C) Selected sequences from Louisiana (indicated in blue) and the United States (indicated in gray) were downloaded from the Global Initiative on Sharing All

Influenza Data website on August 28, 2020. Viral sequences from samples from detained persons residing in dormitories A, D, E, and F are shown in boxes; attempts to perform sequencing on samples obtained from persons in dormitories B and C were unsuccessful. All study sequences cluster together in clade 20C. B and D) Zoomed in branch views of sequenced samples obtained from Facility X. The genome from a Louisiana isolate with the fewest single-nucleotide mutations from the study sequences is shown. The 3 groups formed by genetic relatedness of sequences obtained from persons in Facility X are shown. Panel B depicts whether a sample was obtained from persons who were, or became, symptomatic. Scales in panels A and B indicate sample collection date. Scales in panel C and D indicate number of single-nucleotide mutations from a common ancestor.
